# Transient Expression Assay and Microscopic Observation in Kumquat Fruit

**DOI:** 10.21769/BioProtoc.4968

**Published:** 2024-04-05

**Authors:** Jinli Gong, Xuepeng Sun

**Affiliations:** 1 Collaborative Innovation Center for Efficient and Green Production of Agriculture in Mountainous Areas of Zhejiang Province, College of Horticulture Science, Zhejiang A&F University, Hangzhou, Zhejiang, China; 2 Key Laboratory of Quality and Safety Control for Subtropical Fruit and Vegetable, Ministry of Agriculture and Rural Affairs, Zhejiang A&F University, Hangzhou, Zhejiang, China

**Keywords:** Transient expression, *Agrobacterium tumefaciens*–mediated transformation, Fluorescent protein, Confocal microscopy, Actin cytoskeleton, Citrus

## Abstract

Citrus fruits encompass a diverse family, including oranges, mandarins, grapefruits, limes, kumquats, lemons, and others. In citrus, *Agrobacterium tumefaciens*–mediated genetic transformation of Hongkong kumquat (*Fortunella hindsii* Swingle) has been widely employed for gene function analysis. However, the perennial nature of woody plants results in the generation of transgenic fruits taking several years. Here, we show the procedures of *Agrobacterium*-mediated transient transformation and live-cell imaging in kumquat (*F. crassifolia* Swingle) fruit, using the actin filament marker GFP-Lifeact as an example. Fluorescence detection, western blot analysis, and live-cell imaging with confocal microscopy demonstrate the high transformation efficiency and an extended expression window of this system. Overall, *Agrobacterium*-mediated transient transformation of kumquat fruits provides a rapid and effective method for studying gene function in fruit, enabling the effective observation of diverse cellular processes in fruit biology.

**Graphical overview**


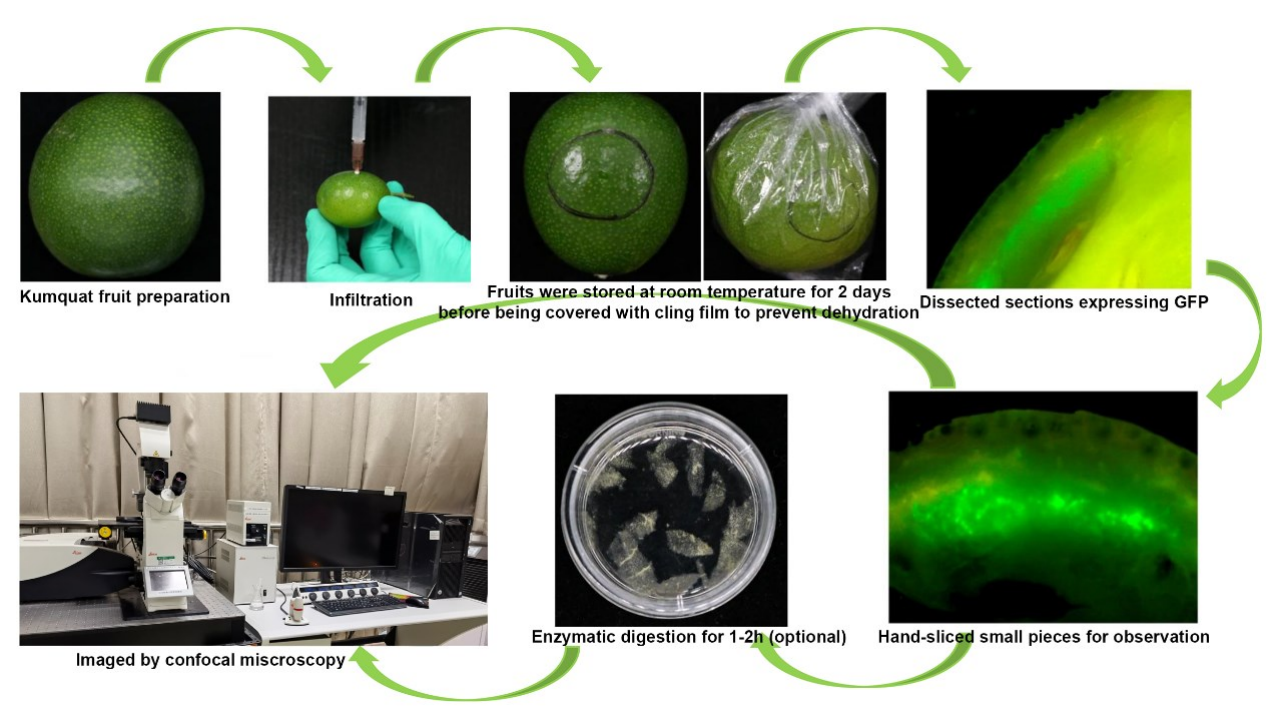


**Schematic illustration of instantaneous transformation system of citrus fruit**

## Background

Citrus, one of the most important fruit crops in the world, is subjected to a variety of environmental stimuli and developmental signals during its growth. Observing the dynamics of organelles may provide new insights into the vital behavior of fruits in response to these developmental and environmental signals. The use of green fluorescent protein (GFP) and its derivatives is a great improvement for cell biological studies, as it can be fused to genes of interest to study their subcellular localization, dynamics, and protein–protein interactions [1]. However, cell structures and subcellular activities from citrus fruits can hardly be observed by light microscopy due to their 3D structure and little transparency. The lack of efficient transformation techniques and the long juvenile phase of citrus plants [2] make the study of cell biology even more difficult. Therefore, developing an effective method for transient transformation of citrus species is important. *Agrobacterium*-mediated infiltration of tobacco leaves was an early-developed method for transient expression [3] and has been widely used to study cell morphology and dynamics. Subsequently, *Agrobacterium-*mediated transient expression methods have been developed for a wide range of leaves and fruits including *Arabidopsis* [4], tomato [5], strawberry [6], apple [7,8], and also citrus fruits [9,10]. Here, the kumquat (*Fortunella crassifolia* Swingle) fruit has been selected for *Agrobacterium*-mediated transient expression. It is a major citrus cultivar in southern China, bearing smaller fruits in the *Citrus* genus compared to pomelo, oranges, and tangerines [11]. It is also characterized by its high sugar content, thin skin, and fewer juice cells, which provides a good environment for infiltration and infection by *Agrobacterium* [12].

In eukaryotic cells, the cytoskeleton is a network of protein–fiber reticulum consisting of microtubules, microfilaments, and intermediate filaments, which not only plays an important role in maintaining cell morphology, withstanding external forces, and maintaining the orderliness of the internal cell structure, but also participates in many important biological processes. Hence, imaging this network in its native and mobile states is of great importance. Here, using the GFP-Lifeact for actin cytoskeleton as an example, we describe methods for *Agrobacterium*-mediated transient transformation of citrus fruit and live-cell imaging in citrus fruit. The high expression of fluorescent protein in kumquat fruit, combined with subsequent live cell imaging, provides a method for studying gene function, subcellular localization, and cellular activity in fruit.

## Materials and reagents

**Biological materials**

1. Kumquat (*F. crassifolia* Swingle) fruit (collected from Guangxi Institute of Citrus Research, located at Guilin city, Guangxi, China)

**Reagents**

1. Actin cytoskeleton marker GFP-Lifeact containing a 35S::GFP cassette as a visual reporter [13]
2. P19 *Agrobacterium*
3. Yeast extract (OXOID, catalog number: LP0021)
4. Tryptone (OXOID, catalog number: LP0042)
5. NaCl (HUSHI, catalog number: 10019318, CAS: 7647-14-5)
6. D-glucose (HUSHI, catalog number: 10010518, CAS: 14431-43-7)
7. MES (Sigma-Aldrich, catalog number: M8250)
8. Na_3_PO_4_ (HUSHI, catalog number: 20040928, CAS: 7601-54-9)
9. Acetosyringone (Sigma-Aldrich, catalog number: D134406)
10. GV3101 Chemically Competent Cell (Shanghai Weidi Biotechnology, catalog number: AC1001)
11. Antibiotics: Kanamycin (Kan) (Genview, catalog number: AK177-10G) and Rifampicin (Rif) (Genview, catalog number: AR280-1G)
12. Antibodies: Polyclonal mouse antibody against GFP (Biorbyt, catalog number: orb688437) as the primary antibody and goat anti-mouse HRP (Biorbyt, catalog number: orb670233) as the secondary antibody
13. Dimethyl sulfoxide (DMSO) (MACKLIN, catalog number: C13178602)
14. Mannitol (Solarbio, catalog number: M8141)
15. KCl (Rhawn, catalog number: R051965)
16. CaCl_2_ (MACKLIN, catalog number: C832203)
17. BSA (MACKLIN, catalog number: B928042)
18. Cellulase R10 (Yakult, catalog number: 220908-02)
19. Sodium hypochlorite (MACKLIN, catalog number: S935360)

**Solutions**

1. Luria Broth (LB) medium (see Recipes)
2. Infiltration buffer (see Recipes)
3. Enzymatic solution (see Recipes)
4. Antibiotics (see Recipes)

**Recipes**

1. **LB media**
  10 g of tryptone, 10 g of NaCl, 5 g of yeast extract; make up to 1 L with dH_2_O, following autoclave sterilization.
2. **Infiltration buffer**
**Infiltration medium stock solutions:**
  500 mM MES: 4.88 g of MES in 50 mL of dH_2_O. Store at 4 °C.
  20 mM Na_3_PO_4_: 0.17 g of Na_3_PO_4 _in 50 mL of dH_2_O. Store at 4 °C.
  1 M Acetosyringone (3’,5’-dimethoxy-4’-hydroxyacetophenone): 0.196 g of Acetosyringone in 1 mL of DMSO. Divide into single-use aliquots and store at -20 °C.
**50 mL infiltration buffer**
  250 mg of D-glucose, 5 mL of MES stock solution, 5 mL of Na_3_PO_4_ stock solution, 5 μL of Acetosyringone stock solution; make up to 50 mL with dH_2_O.
3. **Enzymatic solution**
  Enzymatic solution stock solutions:
  0.8 M mannitol stock: 7.29 g of mannitol in 50 mL of dH_2_O.
  2 M KCl stock: 7.455 g of KCl in 50 mL of dH_2_O.
  0.2 M MES stock: 1.952 g of MES in 50 mL of dH_2_O. Store at 4 °C.
  1 M CaCl_2_ stock: 1.11 g of CaCl_2_ in 10 mL of dH_2_O.
  10% BSA stock: 0.1 g of BSA in 1 mL of dH_2_O.
  10 mL enzymatic solution: 0.15 g of cellulase R10, 0.04 g of macerozyme R1, 5 mL of 0.8 M mannitol stock, 100 μL of 2 M KCl stock, and 1 mL of 0.2 M MES (pH 5.7) stock. Heat solution in heat block or oven set to 55 °C (maximum) for 10 min to help enzymes dissolve (or place in 55 °C water) and then cool to room temperature (RT). Next, add 100 μL of 1 M CaCl_2_ stock, 100 μL of 10% BSA stock, and dH_2_O up to 10 mL. Sterilize by filtration (0.45 μm).
4. **Antibiotics**
  50 mg/L Rif
  50 mg/L Kan

**Laboratory supplies**

1. Tips (Axygen, catalog number: AXYT1000B)
2. 1 mL syringe with needle (GEMTIER, catalog number: 0.45X16 RW LB)
3. Glass microscope slides, coverslips (Corning, catalog number: CLS294875X25, CLS285522), and Vectashield (Vectorlabs, catalog number: H-1000) for microscopy
4. Test tubes (Eppendorf, catalog number: EP0030122178) and flask (Corning, catalog number: CLS4444250) for culturing *Agrobacterium*
5. Fine forceps and razor blades (Artis Tweezer, catalog number: Z742671; smartSlicer, catalog number: Z740503)
6. Marker pen (STATMARK, catalog number: Z648191)
7. Tray (PureSolv, catalog number: Z681687) and Falcon tube cap (Corning, catalog number: 352070) for placing fruit
8. Petri dishes (60 mm, optional, for enzymatic digestion of protoplasts) (Nalgene, catalog number: TMO5921-0060)
9. Pipette (Eppendorf, catalog number: GN686271)
10. Rubber gloves (Microflex, catalog number: Z265179)
11. Bibulous paper (for uptaking the extravasated *Agrobacterium*)
12. 50 mL Falcon tubes (Corning, catalog number: 352070)

## Equipment

1. Shaking incubator (Radobio, model: Stab M1T) used for the growth of *Agrobacterium* at 28 °C
2. Centrifuge (Eppendorf, model: 5424)
3. Clean workbench (AIRTECH, model: SW-CJ-2FD)
4. Fluorescence dissecting stereomicroscope (Olympus, model: SZX7)
5. Confocal microscope (Leica, model: SP8)
6. Nanodrop spectrophotometer (or equivalent) to determine optical density of bacterial culture

## Procedure

- **Preparation of kumquat fruit for infestation**
  1. Carry out transformation of citrus fruit on the kumquat (see Note 1). Harvest “Huapi” kumquats or “Rongan” kumquats 150–210 days after flowering (see Note 2). Sterilize with 2% sodium hypochlorite solution for 2 min and rinse thoroughly in water; then, drain on bibulous paper before *Agrobacterium* injection.
  2. Choose the healthy and fresh fruit for infiltration.
- ***Agrobacterium* preparation**
  1. Incubate *Agrobacterium* strain GV3101 (see Note 3) carrying GFP-Lifeact and P19 in liquid LB medium with Kan and Rif antibiotics overnight at 28 °C with shaking at 220 rpm for 12–16 h, to an optical density (O.D.) over 1.0.
  2. Centrifuge *Agrobacterium* culture in a 50 mL Falcon tube at 5,000× *g* for 5 min to pellet the cells.
  3. Discard the supernatant and resuspend *Agrobacterium* pellet using infiltration buffer.
  4. Spin down the culture at 5,000× *g* for 5 min and discard the supernatant. Suspend the culture with the infiltration buffer to reach a final OD_600_.
  5. Measure the OD_600_ of *Agrobacterium* cells and add appropriate volumes of infiltration buffer to dilute the *Agrobacteria* to the desired O.D. (usually 0.8 O.D., see Note 4). If you want to co-express two or multiple proteins, mix different kinds of *Agrobacteria* with the desired O.D. for each one. Every construct needs to be mixed in equal amounts with P19 *Agrobacterium* (see Note 5).
- ***Agrobacterium* infiltration**
  1. Cut off part of the needle of the syringe, leaving a needle approximately 0.1–0.3 cm in length.
  2. Hold the bottom of the fruit in one hand and gently inject the bacteria solution into the epicarp at a depth of 0.1–0.3 cm. With gentle pressure on the plunger, carefully inject bacteria mixture into the fruit tissues (Video 1). Try to be as gentle as possible when injecting to avoid damaging the fruit. Inject each fruit with 0.2–0.5 mL of infiltration solution (see Note 6). The mixture could potentially distribute to an area with a radius of 0.8–1.3 cm from the injection point (data not shown). The infiltrated area can easily be seen as a dark, water-soaked region.
  3. Get rid of the overflowing *Agrobacterium* on the surface of the fruit with bibulous paper. Mark the injection site with a marker pen to facilitate sampling (Video 1).
    **Video 1.**
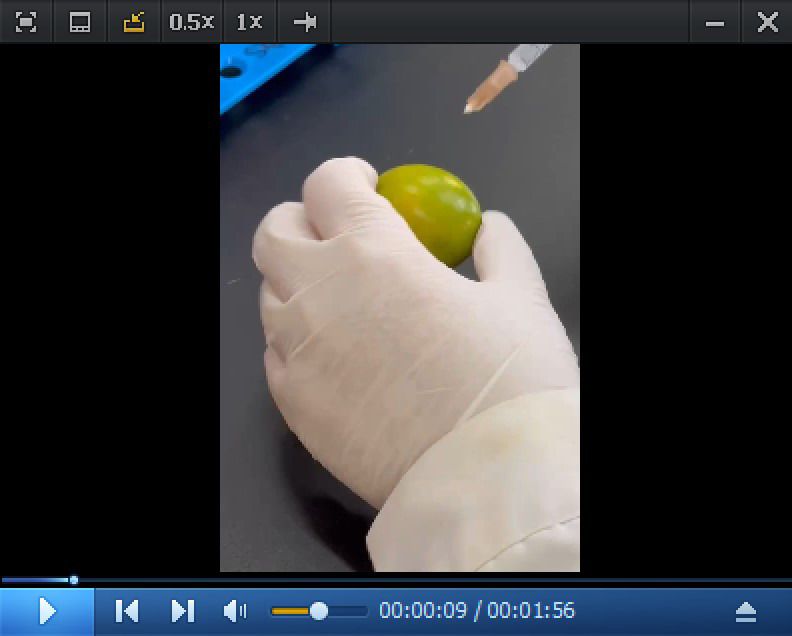
Procedure for fruit injection
  4. Place the infiltrated fruit on the cap of 15/50 mL Falcon tubes and store at RT (25 °C) for approximately two days. Then, wrap with cling film to prevent dehydration (see Note 7) and continue to store at RT.
- **Observation of fluorescent signals**
  Approximately five days after infiltration (see Note 8), swiftly dissect the tissue infiltrated with *Agrobacterium* using a sharp blade (underscored area), with no particular concern for the size or thickness of tissue blocks. Subsequently, place these tissue blocks gently on a microscopy slide and immediately observe under a stereomicroscope equipped with a DP70 camera. Fluorescent signals near the injection site are distinctly visible (see Note 9). The purpose of this step is to preliminarily assess the expression, intensity, and tissue localization of the target protein through fluorescent signals, facilitating subsequent sampling. Detailed subcellular imaging, providing information on cellular localization, necessitates confocal microscopy.
- **Confocal microscopy**
  1. Hand-slice tissues with strong fluorescence signals into small pieces, as thin as possible, and transfer to a slide for confocal microscopy directly (see Note 10).
  2. (Optional if you need to use protoplasts for microscopic observation) Incubate samples for confocal microscopy in sterilized enzymatic solution for 1–2 h in the dark (see Note 11) with shaking at 40 rpm at room temperature to partially digest the cell wall. The main purpose of this additional step is to release the cells from the tissue to facilitate observation. Prolonged digestion can result in the production of protoplasts, but it is normally unnecessary; ideally, it is good to keep any disruptions of this process to a minimum (Observation of Golgi apparatus movement using protoplasts is shown in Video 2).
    **Video 2.**
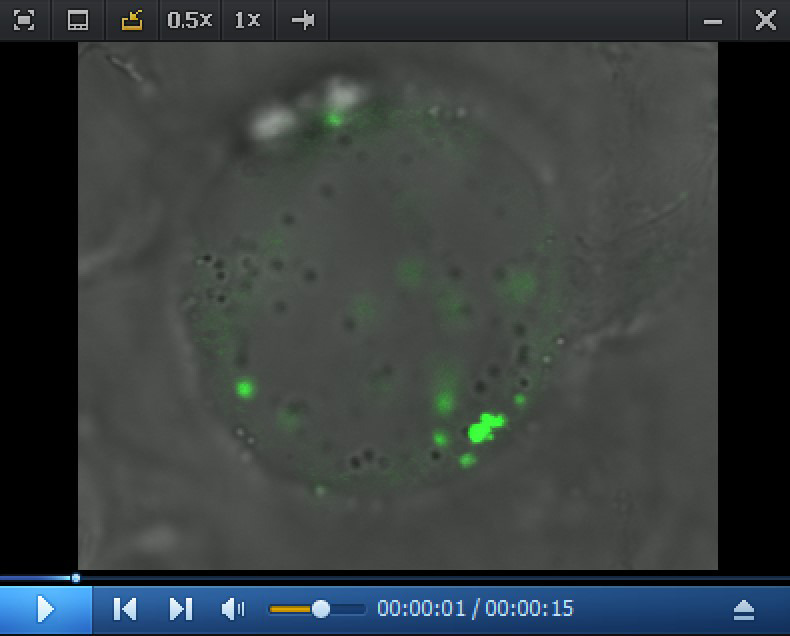
Golgi movement in citrus fruit protoplast
  3. Mount samples in microscope slides and seal with Vectashield; then, press the coverslip gently from one side.
  4. Carry out live-cell imaging using a Leica SP8 laser scanning confocal microscope with a 63× oil immersion lens. For wavelength settings, GFP was excited at 488 nm and detected at 505–550 nm.

## Data analysis

**Figure 1.**
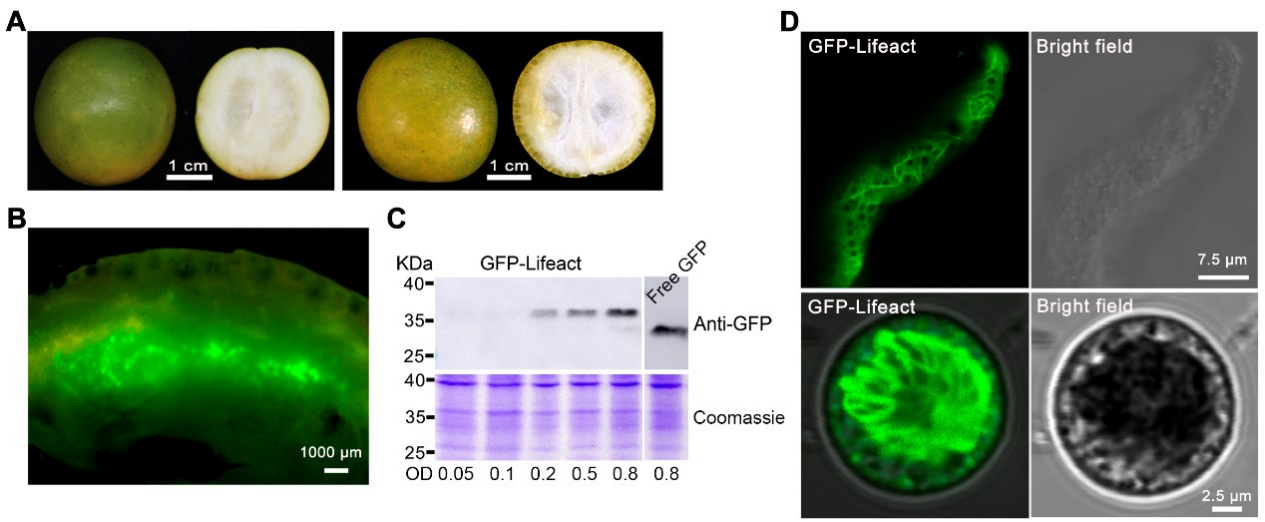
Transient expression of fluorescent protein GFP-Lifeact in kumquat fruit cells. A. Representative image of “Huapi” (left) and “Rongan” (right) kumquat fruit used for injection. B. Dissected sections expressing GFP fusion proteins. C. Western blotting analysis of GFP-Lifeact and free GFP (from pMDC43 binary vector) expression in kumquat fruit using different concentrations of *Agrobacterium* suspension; 20 μg of total protein from each lane were probed with anti-GFP antibodies with 1:2,000 dilution. D. Representative images of fluorescent protein fusions localized to actin cytoskeleton in fruit cells through manual sectioning (up) and protoplasts (down). The construct GFP-Lifeact was infiltrated at OD_600_ = 0.8 and images were taken five days after infiltration.

**Figure 2.**
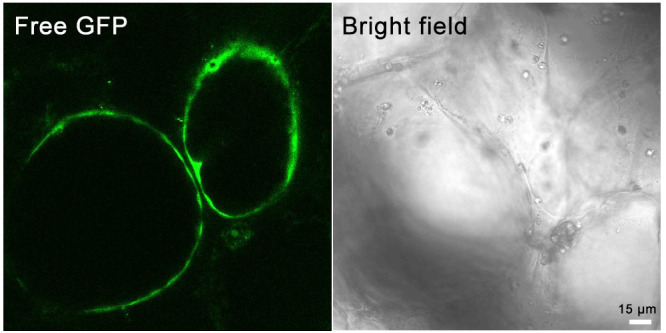
Transient expression of the free GFP in kumquat fruit cells

“Huapi” or “Rongan” kumquat fruits (Figure 1A) were used for the transient overexpression of GFP-Lifeact. Five days after the injection, the injection site was incised, and obvious green fluorescence was visible under a stereomicroscope (Figure 1B). Fluorescent signals could be detected in different tissues of the kumquat, including the flavedo, albedo, juice sacs, and partition, into which the *Agrobacterium* solution penetrated [12]. Western blot analysis conducted on kumquat tissues five days post-injection revealed that the expression of GFP-Lifeact was discernible at all *Agrobacterium* OD values exceeding 0.2. Furthermore, the protein expression level of GFP-Lifeact exhibited enhancement with escalating concentrations of *Agrobacterium* injection (Figure 1C). Tissues exhibiting robust fluorescent signals were meticulously sectioned as thinly as possible, and the GFP-Lifeact-labeled actin cytoskeletons were clearly observed under confocal microscopy through manual sections and protoplasts (Figure 1D). However, the free GFP is ubiquitously distributed throughout the fruit cells, with signals of free GFP detectable in both the membrane and cytoplasm (Figure 2).

## Notes

1. Fruit variety selection: The meticulous choice of fruit varieties holds paramount significance. Kumquat fruits were identified as our preferred material for transient transformation due to their characteristics of thin skin and tender flesh (Figure 1A); additionally, they contain high sugar content and low acid, which provides a suitable growth environment for *Agrobacterium*. Other citrus cultivars, such as orange, pomelo, and mandarin, could also be considered. Notably, kumquat exhibited sustained production of robust signals and high conversion efficiency (data not shown).
2. Determination of the fruit stage: The mature green stage of kumquat fruit (approximately 150–210 days after flowering) has proven to be optimal for transient transformation, owing to its firm texture, heightened metabolic activity, and finer peels. Additionally, post-harvest kumquat fruit remains suitable for experimentation, underscoring the importance of selecting fresh citrus fruits for injection in subsequent procedures. It is advised to avoid fruit at the end of the ripening stage, as it is susceptible to rot following *Agrobacterium* injection.
3. Strain selection: Various strains of *Agrobacterium tumefaciens* (e.g., EHA105 or GV3101) were selected for testing. The findings revealed that transformation mediated by GV3101 yielded the highest protein output.
4. Optical density value: Attaining the optimal *Agrobacterium* density (OD_600_) for each construct is crucial, and this requirement varies significantly from gene to gene. The OD_600_ of *Agrobacterium* also exerts an impact on the level of protein expression, necessitating the optimization of the ideal OD_600_ for a new construct prior to the commencement of the actual study. An OD_600 _concentration of *Agrobacterium* below 0.1 typically results in weak expression, while an OD_600_ exceeding 1.0 tends to induce yellowing and rotting of fruit. If mitigating potential overexpression artifacts is a primary concern, it is advisable to use the lowest O.D. that still yields sufficient signal. Otherwise, an OD_600_ within the range of 0.5–0.8 can be employed to achieve maximum expression.
5. P19: Augmented expression can be attained through the utilization of p19 constructs, effectively averting gene silencing.
6. Injection dose: Maintaining the injection volume within the range of 0.2–0.5 mL for each fruit is imperative, as excessive infiltration buffer may lead to unforeseen rotting.
7. Bagging: After two days of fruit injection, it is advisable to individually encase fruits with cling film to enhance water retention and ensure air permeability; bagging should not occur too early, otherwise it will cause fruit rot due to high humidity.
8. Time of expression: Different proteins may exhibit distinct expression timelines. Generally, protein expression becomes detectable 4–5 days after infiltration. If the fruit preservation conditions are favorable, protein expression can even be detected up to one month post-infiltration. The persistence and abundance of protein expression typically depend on the plasmid itself and the vitality of the fruits.
9. Fruit tissues for expression: Fluorescent signals can be observed in various tissues of kumquat, encompassing flavedo, albedo, and juice sacs, where the *Agrobacterium* solution can penetrate. The efficacy of expression is contingent upon the uptake of *Agrobacterium* by different tissues, with superior absorption correlating to enhanced expression strength. Typically, the expression of fluorescent proteins in albedo surpasses that in other tissues.
10. During the process of slicing fruit tissue, it is imperative to minimize external pressure applied to the sample. The use of a sharp razor blade is equally crucial as it mitigates mechanical damage and reduces the risk of fractures.
11. Enzymolysis: The primary objective of this supplementary step is to release protoplasts from tissues to facilitate observation. Although prolonged digestion could yield protoplasts, it is generally unnecessary, and the ideal approach is to minimize any disruptions.

## Validation of protocol

This protocol has been used and validated in the following published articles:

1. Gong, J. L. et al. (2021). Illuminating the cells: transient transformation of citrus to study gene functions and organelle activities related to fruit quality. Hortic Res 8(1): e1038/s41438–021–00611–1, DOI: 10.1038/s41438-021-00611-1.
2. Gong, J. L. et al. (2021). Red light-induced kumquat fruit coloration is attributable to increased carotenoid metabolism regulated by FcrNAC22. Journal of experimental botany 72: 6274–6290, DOI: 10.1093/jxb/erab283.
